# Understanding the Molecular Mechanism of Interventions in Treating Rheumatoid Arthritis Patients with Corresponding Traditional Chinese Medicine Patterns Based on Bioinformatics Approach

**DOI:** 10.1155/2012/129452

**Published:** 2012-10-16

**Authors:** Miao Jiang, Cheng Lu, Gao Chen, Cheng Xiao, Qinglin Zha, Xuyan Niu, Shilin Chen, Aiping Lu

**Affiliations:** ^1^Institute of Basic Research in Clinical Medicine, China Academy of Chinese Medical Science, Beijing 100700, China; ^2^School of Life Science, Hubei University, Wuhan, Hubei 430062, China; ^3^Institute of Clinical Medicine, China-Japan Hospital, Beijing 100029, China; ^4^National Research Center of TCM, Jiangxi University of Chinese Medicine, Nanchang, Jiangxi 330006, China; ^5^Institute of Medicinal Plant Development, Chinese Academy of Medical Sciences, Beijing 100094, China; ^6^School of Chinese Medicine, Hong Kong Baptist University, 7 Baptist University Road, Kowloon Tong, Kowloon, Hong Kong

## Abstract

Better effectiveness would be achieved when interventions are used in treating patients with a specific traditional Chinese medicine (TCM) pattern. In this paper, the effectiveness in treating rheumatoid arthritis (RA) patients in a randomized clinical trial as reanalyzed after the patients were classified into different TCM patterns and the underlying mechanism of how the TCM pattern influences the clinical effectiveness of interventions (TCM and biomedicine therapy) was explored. The pharmacological networks of interventions were builtup with protein and protein interaction analyses based on all the related targeted proteins obtained from PubChem. The underlying mechanism was explored by merging the pharmacological networks with the molecular networks of TCM cold and hot patterns in RA. The results show that the TCM therapy is better in treating the RA patients with TCM hot pattern, and the biomedical therapy is better in the RA patients with cold pattern. The pharmacological network of TCM intervention is merged well with the molecular network of TCM hot pattern, and the pharmacological network of biomedical therapy is merged well with the network of cold pattern. The finding indicates that molecular network analysis could give insight into the full understanding of the underlying mechanism of how TCM pattern impacts the efficacy.

## 1. Background

Traditional Chinese medicine (TCM) is an essential part of the health care system in several Asian countries, and is considered a complementary or alternative medical system in most western countries [1]. The TCM pattern (also called Zheng or Syndrome differentiation) is the basic unit in the diagnostic system of TCM. The pattern is determined by analyzing the patient's overall signs and symptoms, including tongue appearance and pulse manifestation [2, 3]. Currently, diagnoses based on the TCM pattern are integrated with biomedical diagnosis, and the integrative medicine model has become common in clinical practice [4]. TCM pattern classification has proven to be an effective method in patient stratification integrated with biomedical diagnostic method. 

Our previous study has reported that the molecular networks of major TCM patterns, cold and hot pattern in rheumatoid arthritis (RA), has been built-up by network analysis [5]. Also we reported that, in an RCT clinical trial, biomedicine therapy was better than the Chinese herbal therapy in the treatment of RA with regard to the ACR responsive rate [6]. We presume that the effectiveness of the two interventions would be different if the RA patients were divided into TCM cold and hot patterns, and the molecular mechanism about the different effectiveness of the two interventions can be explored by network pharmacology method based on the molecular networks of TCM cold and hot pattern in RA [7]. In this paper, randomized clinical data were re-analyzed with focus on the comparison of effectiveness of two interventions (a TCM therapy and a biomedicine therapy) in treating RA patients who were classified into TCM cold and hot patterns, and the molecular mechanism involved in the different effectiveness of two interventions was explored by merging the network pharmacology of the interventions and the molecular networks of TCM patterns in RA. The pharmacological networks of the interventions can be built-up as previously reported [8], and the molecular networks of cold and hot patterns in RA have been built-up previously [5].

## 2. Materials and Methods

### 2.1. Briefings about the Reported Clinical Trial

A randomized, controlled clinical trial at 9 centers throughout China was conducted according to the guidelines of the Declaration of Helsinki and the principles of Good Clinical Practice. Details about the clinical trial have been reported previously [9]. Patients were randomly assigned in a 1 : 1 ratio to receive a biomedical combination therapy or TCM therapy. Patients, men and women, aged from 18 to 70 years old were eligible to participate if they met the American College of Rheumatology (ACR) criteria for RA, had disease duration of at least one year, and were categorized as Class I, II, or III under ACR guidelines [10]. Patients receiving nonsteroidal antiinflammatory drugs, corticosteroids (up to 10 mg per day), or both were allowed to participate in the trial if they had been on stable doses for at least four weeks prior to randomization. Patients with severe cardiovascular, lung, liver, kidney, hematologic, or mental diseases and women who were pregnant, breast-feeding or planning to become pregnant in the next 8 months were excluded. An Institutional Review Board approved the protocol at each center. Each patient gave the written informed consent prior to any study-related procedures. Biomedical combination therapy included use of nonsteroidal antiinflammatory drugs (NSAIDs) and disease modifying antirheumatic drugs (DMARDs). Diclofenac (extended action 75 mg tablet), the NSAID given, was taken once a day orally after a meal but was discontinued after the acute inflammation was controlled. The DMARDs used were methotrexate and sulfasalazine. Methotrexate (MTX) was taken orally once a week at a starting dosage of 5 mg with addition of 2.5 mg each week up to 15 mg, and a maintenance dosage from 2.5 mg to 7.5 mg. Sulfasalazine (SSZ) was taken orally twice a day from a starting dosage of 0.25 g with the addition of 0.25 g each week, to a maintenance dosage of 0.5 g to 1 g, four times a day. Chinese herbal medicine therapy (TCM therapy) was recommended by Chinese Association of Traditional Chinese Medicine and clinical reports [11], and all patients took Glucosidorum Tripterygll Totorum (produced by GMP certificated Hubei Huangshi Pharmaceuticals of China), which contains the Chloroform extract (mainly glycosides) of Glucosidorum Tripterygll Totorum, 10 mg each time, three times a day, and Yishen Juanbi Tablet (produced by GMP certificated Qingjiang Pharmaceuticals of China), which contains the water extracts of herbs including Radix Rehmanniae, Radix *Angelicae sinensis*, *Herba epimedii*, Rhizoma drynariae, *lumbricus*, Nidus vespae, *Eupolyphaga seu steleophaga*, 8 g (equal to 50 g raw herbal materials) each time, three times a day, taken orally after meals.

Within 14 days before randomization, a complete physical examination, medical history, and assessment of the symptoms typically evaluated by TCM physicians were performed and recorded. After treatment for 24 weeks, the ACR 20 response was evaluated as the efficacy [10], and the evaluation was done by a third party (rheumatologists at each center) who did not know the therapy. An ACR 20 response is defined as a reduction of at least 20 percent of the number of tender joints and swollen joints plus an improvement of at least 20 percent in at least three of the following five criteria: patient's assessment of pain, patient's assessment of disease activity, physician's assessment of disease activity, patient's assessment of physical function, and serum C-reactive protein concentration.

Data pertaining to the symptoms used to determine TCM were recorded at the patient's entry into the study and every two weeks thereafter until week 24 [6]. The 13 symptoms, which are commonly used for TCM pattern classification for RA patients [6], were assessed, and they are cold feeling in whole body, cold feeling in joints, cold feeling in limbs, lassitude, heavy feeling in limbs, numbness in limbs, vexation, feverish joint, thirst, nocturia, dizziness, turbid yellow colored urine, and fever. The symptoms were scored at four strength levels by patients as none, slight, medium, or severe and were recorded as 0, 1, 2, or 3, respectively. In addition, tongue appearance such as the color of tongue body and tongue fur was also recorded. In this paper, the RA patients were divided into TCM cold and hot patterns with latent class analysis (LCA) method, which can divide the RA patients into different classes based on the symptoms collected. 

### 2.2. Pharmacological Network Building-Up for the Interventions

The pharmacological networks of the interventions were built-up as previously reported [8]. In brief, the information about all herbs (in the TCM intervention) related genes, diseases, TCM effects, and TCM ingredients automatically were mined by using TCMGeneDIT [12], a database system containing a vast amount of biomedical literature. The information on human protein-protein interaction (PPI) networks of biomedical combination (MTX and SSZ combination) and the TCM therapy, and PPI network information about RA were obtained from databases, including PubMed, BIND (Biomolecular Interaction Network Database), BioGRID (The General Repository for Interaction Datasets), DIP (Database of Interacting Proteins), HPRD (Human Protein Reference Database), IntAct (Database system and analysis tools for protein interaction data) and MINT (Molecular Interactions Database), and complemented with curated relationships parsed from Literature using Agilent Literature Search. The *t* value was calculated according to the previously reported [12, 13] and a *t* value greater than 95% was taken as significant information. If the *t* value is larger than 2.576, 2.326, 1.960, or 1.645, we can reject the null hypothesis with 99.5%, 99%, 97.5%, or 95% confidence, respectively. The PPI network was visualized using cytoscape software [14]. The IPCA algorithm was employed to analyze the characteristics of the network, which can detect densely connected regions in the interactome network [15]. Interactomes with a score greater than 2.0 and at least four nodes were taken as significant predictions.

In order to identify the function of each cluster generated by IPCA individually, GO clustering analysis was performed with the proteins described in all subnetworks. For this purpose, the latest version of Biological Network Gene Ontology (BiNGO) tool [16] was used to statistically evaluate groups of proteins with respect to the existing annotations of the Gene Ontology Consortium. The degree of functional enrichment for a given cluster was quantitatively assessed (*P* value) by hypergeometric distribution and implemented in the BiNGO tool. BiNGO currently provides statistical tests for assessing the enrichment of a GO term in the test set, and also provides an approximate *P* value [16]. We selected the 5 GO biological categories with the smallest *P* values as significant.

### 2.3. Merging of Pharmacological Networks of the Interventions with the Molecular Networks of TCM Cold and Hot Patterns in RA

The molecular networks of cold and hot patterns in RA were reported previously [5], and the networks were used to be merged with the pharmacological networks of interventions built-up in this paper. Graph was used merge tool to find the union, intersection, and difference of networks based on node identifiers/IDs. The nodes were matched according to ID mappings, that is, the nodes whose IDs were mapped to each other were considered as the same node when the value of their selected attribute(s) matchs, and then the attribute of source networks is merged into attributes in the resulting network. 

### 2.4. Statistics

All data were analyzed on a SAS9.1.3 statistical package (order no. 195557). TCM pattern was defined by factor analysis based on the loadings on all collected 13 symptoms, and the effective rates in patients with TCM pattern changes were analyzed with the Chi-square method. LCA was employed to explore the exact indications of the interventions. LCA was obtained at http://methodology.psu.edu/index.php/ra/lcalta. In LCA, the latent variable is discrete, and a population is divided into mutually exclusive and exhaustive categories.

## 3. Results

### 3.1. ACR 20 Response (Effectiveness) Evaluation

In this clinical trial, 194 patients treated with biomedical combination therapy and 204 cases treated with TCM therapy finished a whole 24 week treatment, and the ACR 20 responsive rates of biomedical combination therapy and TCM therapy at 24 weeks were 69.59% and 53.20%, respectively. Biomedical combination therapy was more effective than TCM therapy (*P* < 0.05).

**Figure 1:**
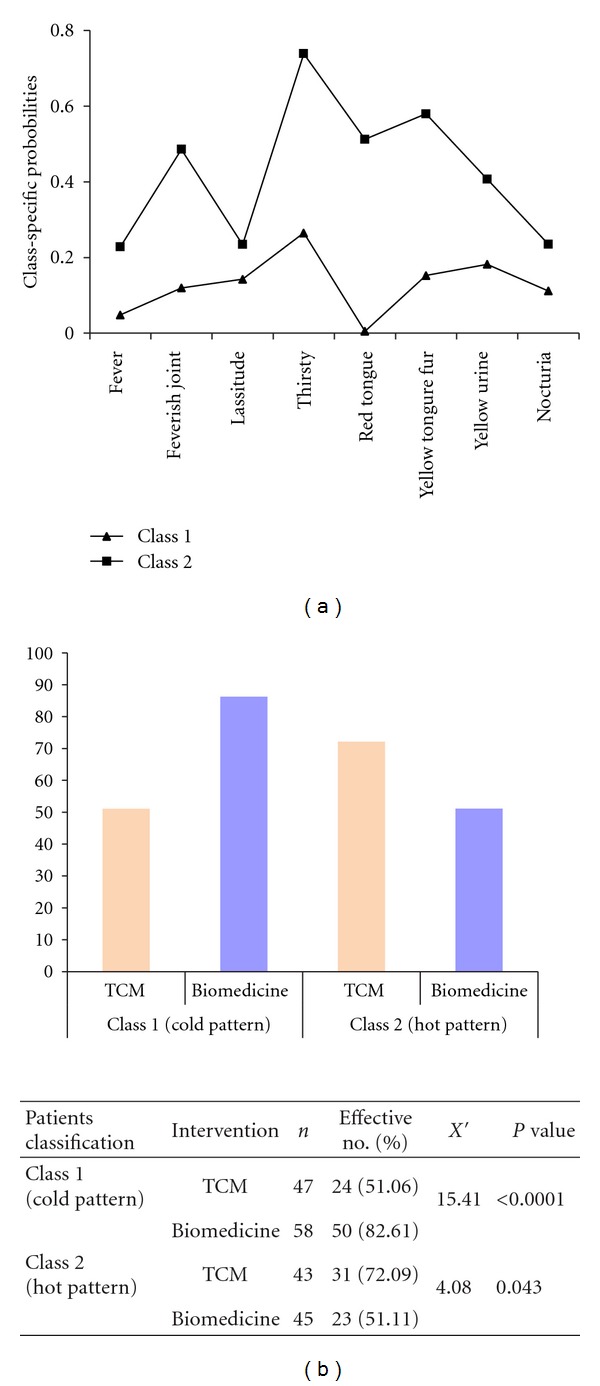
The responsive rates of biomedicine therapy and TCM therapy in treating RA patients with TCM cold and hot patterns. (a) shows two latent classes obtained. Class 1 includes less probability of fever, feverish joints, lassitude, thirst, red tongue, yellow tongue fur and turbid yellow colored urine, which is similar to the clinical manifestations of cold pattern. Class 2 shows higher frequency of those clinical manifestations, which is similar to clinical manifestations of hot pattern. (b) shows the ACR 20 responsive rates of TCM and biomedicine interventions in each latent class of patients. The responsive rate of TCM in RA patients with hot pattern was 72.09%, yet the corresponding rate of biomedicine was only 51.11% (*P* = 0.043 < 0.05). The responsive rate of TCM in RA patients with cold pattern was 51.06%, much lower than that of biomedicine therapy (82.61%, *P* < 0.001).

In the patient classification into TCM cold and hot patterns, an LCA model was established and the TCM information was divided into 2 latent classes according to the clinical responses. class 1 patients had clinical manifestations similar to the cold pattern, including less probability of fever, feverish joints, lassitude, thirst, red tongue, yellow tongue fur and turbid yellow colored urine. Class 2 patients had clinical manifestations similar to the hot pattern, with higher probability of the aforementioned symptoms (Figure 1(a)). ACR 20 responsive rates of interventions in each latent class patients are presented in Figure 1(b). The ACR 20 responsive rate of TCM therapy in hot pattern RA patients was 72.09%, yet the responsive rate of biomedicine therapy was only 51.11% (*P* = 0.043 < 0.05), while the responsive rate of biomedicine therapy in cold pattern RA patients was 82.61%, and the responsive rate of TCM therapy was only 51.06% (*P* < 0.0001). The LCA results indicated that biomedicine therapy was promising in cold-pattern whereas TCM therapy was preferable in hot-pattern RA.

### 3.2. Network Based Molecular Mechanism of Biomedicine Therapy in Treating RA Patients

**Figure 2:**
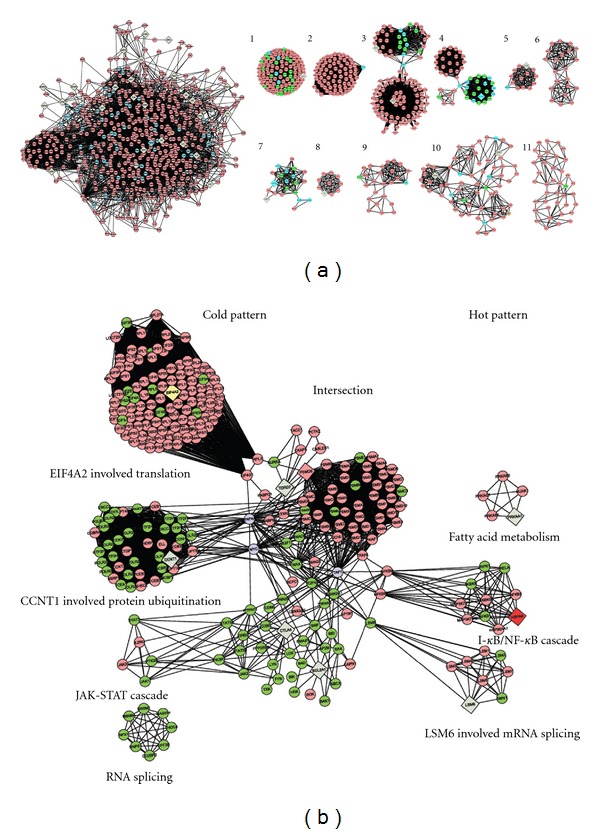
Molecular network based mechanism of biomedicine therapy in treating RA. (a) shows the pharmacological networks of MTX + SSZ obtained with PPI analysis. Node represents protein; edge represents interaction. Left: whole pharmacological network of MTX + SSZ. Right: 11 highly connected clusters were obtained from PPI analysis, and the correlated pathways were shown in Table 1. The green nodes represent the proteins which could be targeted by either MTX or SSZ, or by the interaction between MTX and SSZ. (b) shows the merged networks of MTX + SSZ pharmacological networks and RA pattern networks. Node represents protein; edge represents interaction. Left part is for cold pattern, and right part for hot pattern. The dots between cold and hot pattern are the intersection between the two TCM patterns. The green nodes represent the proteins which could be targeted by either MTX or SSZ, or by the interaction between MTX and SSZ.

Pharmacological network of the biomedical combination therapy was built up by integrating text mining and protein-protein interaction (PPI) approaches. Text mining results indicated that MTX and SSZ associated with 66 and 77 genes, respectively (data not shown). The intersection network between MTX and SSZ networks was obtained using the graph merge tool (Figure 2(a), left). Eleven highly connected regions were detected by IPCA algorithm, allowing the inference of significant complexes or pathways in this network (Figure 2(a), right). The most relevant functions and pathways extracted from these subnetworks by the Biological Network Gene Ontology tool were related to the ATP biosynthetic process, RNA metabolic process, and regulation of the immune response to viruses, regulation of cell cycle, ATP synthesis, and coupled electron transport (Table 1). This suggests that synergistic mechanisms of MTX + SSZ are involved in regulation of the immune response to viruses, proliferation and apoptosis of T and B cells, and cAMP and NF-*κ*B signaling pathway. Then we combined the cold- and hot-pattern RA networks, which have been built-up previously [5], and the pharmacological networks of biomedicine therapy by using a graph merge tool (Figure 2(b)). In Figure 2(b), all nodes and lines in background represent RA molecular networks. Nodes represent proteins and edges denote interactions. The left part shows the network of the cold pattern of RA, the right part shows that of the the hot pattern. The blue nodes represent the proteins that are targeted by either MTX or SSZ, and the green nodes represent the proteins that are targeted by both MTX and SSZ, or by the interaction between MTX and SSZ. Obviously, biomedicine therapy targets parts of all four cold-pattern clusters (left of Figure 2(b)): regulation of translation, protein ubiquitination pathway, JAK-STAT cascade, and RNA splicing. However, only parts of 2 hot-pattern clusters (right of Figure 2(b)): I-kappa B kinase/NF-kappa B and mRNA splicing were targeted by MTX and SSZ, and fatty acid metabolism involved in hot-pattern RA cannot be targeted by MTX and SSZ, and splicing of mRNA in hot-pattern RA was also targeted only on 2 nodes. The results suggest that the involved pathways in the cold or hot pattern of RA can partly explain why biomedicine therapy appeared to be more effective in treating cold-pattern RA patients from the view of molecular networks.

### 3.3. Molecular Mechanism of the TCM Therapy in Treating RA

**Figure 3:**
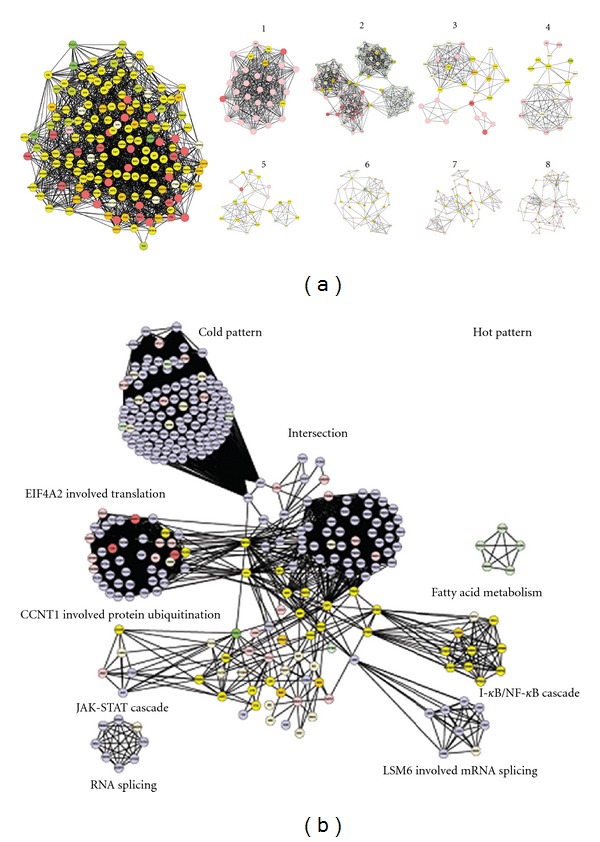
Molecular network based mechanism of TCM therapy in treating RA. (a) shows the pharmacological network of TCM therapy obtained with PPI analysis. Node represents protein, and edge represents interaction. Left: centrality of pharmacological network of TCM therapy. Right: 8 highly connected clusters were obtained from PPI analysis, and the correlated pathways were shown in Table 2. Greed colored nodes represent nodes affected by either Glucosidorum Tripterygll Totorum tablet or Yishen Juanbi Tablet, yellow nodes represent nodes affected by the interaction of Glucosidorum Tripterygll Totorum tablet and Yishen Juanbi Tablet. Red colored nodes represent nonaffected nodes. (b) shows the merged networks of TCM intervention pharmacological networks and RA pattern networks. Node represents protein, and edge represents interaction. Left part is for cold pattern, and right part is for hot pattern. The dots between cold and hot patterns are the intersection between the two TCM patterns. The red and green colored nodes represent the proteins which could be targeted by one of the TCM products, and the yellow nodes represent the proteins which could be targeted by both TCM products, or by the interaction between the two TCM products.

The pharmacological networks of TCM therapy were built up by a similar method to biomedicine therapy. In Figure 3(a), eight highly connected regions were detected by the IPCA algorithm, allowing the detection of significant complexes or pathways in the network. The most relevant functions and pathways extracted from these subnetworks by the Biological Network Gene Ontology tool were related to ATP biosynthetic processes, RNA metabolic processes, regulation of the cell cycle, ATP synthesis, fibroblast growth factor receptor signaling pathway, and Ras protein signal transduction (Table 2). Then we used the graph merge tool to merge the TCM pharmacologic networks with the RA pattern networks (Figure 3(b)). In Figure 3(b), all nodes and lines in the background represent RA molecular networks. Nodes represent proteins and edges represent interactions. The cold pattern of RA is shown on the left, and the hot pattern is shown on the right. The red and green nodes represent the proteins that may be targeted by one herbal product in the TCM therapy, and the yellow nodes represent proteins that could be targeted by both herbal products in the TCM therapy or by the interaction between the two herbal products in the TCM therapy. Clearly, TCM therapy could target all three hot-pattern clusters (shown on the right side: I-kappa B kinase/NF-kappa B, and mRNA splicing), and a small part of the 4 cold-pattern clusters (shown on the left side: regulation of translation, protein ubiquitination pathway, JAK-STAT cascade, and RNA splicing), as well as a majority of nodes in the intersection region. The results suggest that the involved pathways in the TCM therapy may partly explain why the TCM therapy was more effective in treating hot-pattern RA patients.

### 3.4. Effectiveness of the Interventions on the Patients with TCM Pattern Change

TCM pattern can vary following the changes of symptoms. The 13 TCM symptoms in all patients were analyzed with factor analysis to define the TCM pattern changes. In Table 3, Cold feeling in the whole body, cold feeling in the limbs, and cold feeling in the joints were classified as factor 1. Thirst, feverish joints, turbid yellow colored urine, and fever were classified as factor 2. Then the factor scores for each patient were calculated according to the factor loadings of symptoms. Based on the cluster of symptoms for TCM pattern classification, if the patient's factor 1 score was >1 and factor 2 score was <1, the patient was classified as factor 1 pattern (similar to typical hot pattern in TCM), and if the patient's factor 1 score was <1 and factor 2 score was >1, the patient would be classified as factor 2 pattern (similar to typical cold pattern in TCM). In this study, in order to simplify the TCM pattern and easily analyze the TCM pattern change during the therapeutic process, if the patient's factor 1 score was > or =1, the patient was classified as factor 1 pattern (cold dominant pattern, or cold mixed with hot pattern), and other patients with factor 1 score <1 would be classified as noncold dominant pattern (they might be or might not be with hot pattern). 

**Figure 4:**
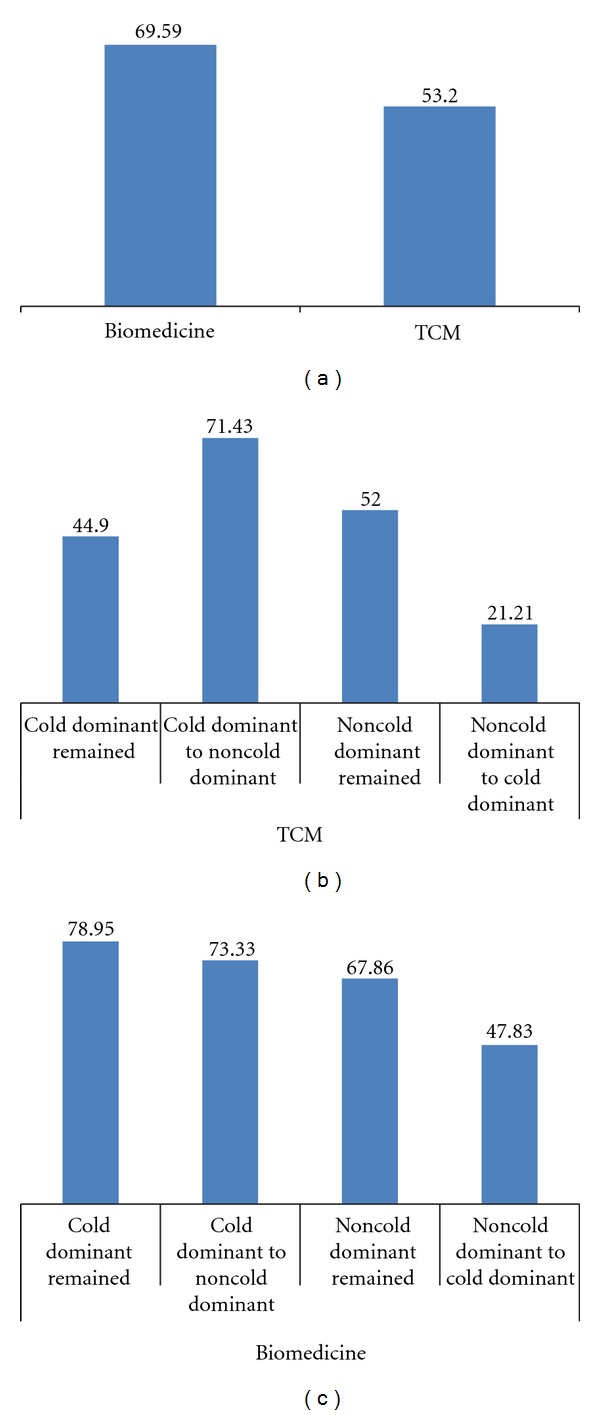
Effective rates of biomedical combination and TCM therapy for RA treatment. (a) effective rate at 24 weeks was 69.59% and 53.20% treated with biomedical combination therapy and TCM therapies, respectively. (b) and (c) ACR 20 response (effective rate) change following the TCM pattern change in TCM and biomedical group. The effective rate of the TCM therapy in the patients who showed TCM pattern changes from cold dominant pattern to noncold dominant pattern was higher (*P* < 0.05). The effective rate in the patients who showed TCM noncold dominant pattern to cold dominant pattern was lower (*P* < 0.05). After biomedical treatment for 24 weeks, the effective rate in the patients who showed TCM cold dominant pattern to noncold dominant pattern was similar to those continuing with cold dominant pattern (*P* > 0.05), while the effective rate in the patients who showed TCM pattern changes from noncold dominant pattern to cold dominant pattern was lower (*P* < 0.05).

Based on the criteria for TCM pattern classification, 115 patients were identified as having TCM cold dominant pattern and 99 patients as noncold dominant pattern in TCM treated patients, and 87 patients were identified as having TCM cold dominant pattern and 107 patients as noncold dominant pattern in biomedicine treated patients. Six months after the treatment, the TCM pattern changed in some patients (Table 4).  Figure 4 shows the ACR 20 response (effective rate) change following the TCM pattern change in TCM and biomedicine group. 

The upper part of Figure 4 shows that, after treatment for 24 weeks, the effective rate of the TCM therapy in the patients who showed TCM pattern changes from cold dominant pattern to noncold dominant pattern was higher (*P* < 0.05). The effective rate in the patients who showed TCM noncold dominant pattern to cold dominant pattern was lower (*P* < 0.05). The Lower part of Figure 4 shows that, after treatment for 24 weeks, the effective rate in the patients who showed TCM cold dominant pattern to noncold dominant pattern was similar to those continuing with cold dominant pattern (*P* > 0.05), though the effective rate of the biomedical combination therapy in the patients who showed TCM pattern changes from noncold dominant pattern to cold dominant pattern was lower (*P* < 0.05); the sample size was too small (23 cases) to yield the conclusion.

### 3.5. Molecular Mechanism for the Effect of Two Interventions in RA Patients with TCM Pattern Changes

**Figure 5:**
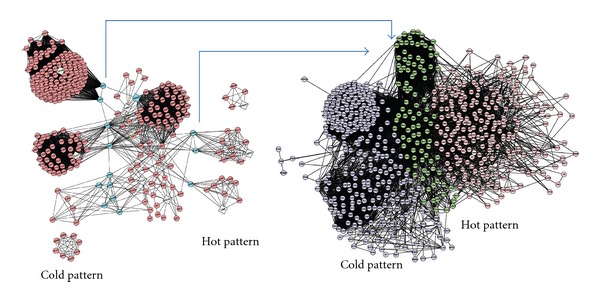
The PPI networks of functional relationships between cold pattern and hot pattern in RA. Diamonds represent seed node. Cycles represent neighbor nodes. All edges represent interactions. Left: Cold pattern network is in the left, and hot pattern is in the right. The middle part is the commonly shared network by both cold pattern and hot pattern and is the common network of RA. Blue colored nodes (between cold pattern or hot pattern and the common shared part) are the connection between TCM pattern related highly condensed networks and common sharing network, which were important for intersection between cold pattern and hot pattern. Right: Further extensive analysis for the intersection between cold and hot patterns in RA.

Firstly the intersection (b)etween the cold and hot patterns was further analyzed (Figure 5) and the major pathways involved in the intersections included the protein ubiquitin pathway (Table 5). Then, we checked back the major pathways induced by the biomedical combination therapy and the TCM therapy (Tables 1 and 2). It was found that the regulation of ubiquitin-protein ligase activity during mitotic cell cycle was the pathway affected by MTX + SSZ combination therapy (Table 1), and no similar pathway can be affected with the TCM therapy (Table 2). These results suggest that the efficacy of biomedical intervention was less likely influenced by TCM pattern changes, while the efficacy of the TCM therapy was most likely influenced by TCM pattern change. The explanations were supported in our clinical data (Figure 4). However, TCM pattern transition is complicated, and more data are warranted for further clarification.

## 4. Discussion 

The major finding in this paper is that it is the first time to clarify the molecular mechanism of interventions in treating RA patients with corresponding TCM cold and hot patterns. This study further suggests that TCM pattern classification can help stratify and tailor the patients for a specific intervention and thus improve the effectiveness of the intervention. 

Pattern differentiation in TCM is based primarily on the patient's symptoms [4, 17]. TCM symptoms assessed in RA patients include those unrelated to the musculoskeletal system. TCM pattern would determine the selection of herbal formula, and previous study also showed that the effective rate of biomedical intervention was higher in treating RA patients with TCM cold pattern [18], which indicates that TCM pattern classification can be regarded as a complementary diagnostic method for patient stratification. A full understanding of the mechanism of TCM pattern expression could aid in our elucidation of the pathogenesis of disease and facilitate the development of improved therapeutic regimens. However, it is very difficult to conduct studies on TCM pattern expression using existing conventional methods [4], because TCM pattern expression is a complicated system, consisting of various manifestations that are seemingly independent from currently accepted biomedical mechanisms. Recent advances in the “-omics” (proteomics, genomics, etc.), bioinformatics, and systems biology have provided us an opportunity to integrate multidimensional data. Bioinformatics and systems biology approaches may open the way to a new convergence of TCM and biomedical approaches in both concept and methodology. Our previous studies have shown the biological networks of RA patients with TCM cold or hot pattern [5], and the network of TCM pattern would be helpful for elucidating the mechanism of intervention in treating RA patients with corresponding TCM patterns. 

The therapeutic mechanism of an intervention is in fact a biological network comprising hundreds or even thousands of gene expressions, which vary between different tissues and effector cells. Systems biology approaches predict biological networks and can lead to a superior insight of biological systems as a whole. Our previous results about the analysis on *Salvia miltiorrhiza* (SM) and *Panax notoginseng* (PN) in combination (SMPN) demonstrated that the therapeutic mechanism of SMPN was likely to associate with proliferation and apoptosis of endothelial cell, apoptosis of arterial smooth muscle cell, apoptosis and regulation of the immune system process within macrophages during foam cell formation, and cardiocyte apoptosis [7]. For other herbal formulae, the pharmacological networks were established with similar methods [19, 20]. In regard to even a pure compound, its pharmacological activity could be explained with a molecular network, and a study showed that Integrin *α*2*β*1 might be one of the direct target proteins of salvianolic acid B (SB) in platelets; the signal cascades network of SB after binding with integrin *α*2*β*1 might include regulation of intracellular Ca²^+^ level, cytoskeleton-related proteins such as coronin-1B, and cytoskeleton structure of platelets [21]. In this study, we built up the networks of TCM herbal formula and the biomedical combination therapy, and merged them with the TCM pattern networks in RA patients. The results at least clarify partially the molecular mechanism on the two interventions in treating RA patients with TCM cold or hot pattern. 

Symptoms vary during the treatment; therefore, TCM patterns in RA patients might change over time. In the clinical trial, all therapies were fixed for the RA patients who met the inclusion criteria. The data in this paper is analyzed retrospectively, and statistical factor analysis was used to divide the RA patients into different groups based on their symptoms obtained at the beginning of the trial, then the patients were classified into two groups with cold or hot pattern for the general ACR 20 responsive rate calculation. Similarly we identified those patients into TCM patterns based on the information before and after the treatment, and pattern changes in some cases. In order to explore the mechanism underlying the efficacy changes of the interventions followed by the TCM pattern changes, the intersection (b)etween TCM cold and hot patterns in RA patients was analyzed with PPI method in the view of molecular network, and then the pathways involved in the intersection network were compared with the pathways involved in the pharmacological network of the two interventions for the mechanism exploration. All together, the results suggest that molecular network analysis can be used to clarify the underlying molecular mechanism of intervention in treating RA patients with different TCM patterns, and also the results further support that TCM pattern classification (including pattern change) could be useful in stratifying RA patients for the improvement of the intervention. Further RCTs with TCM pattern identification at beginning were needed.

Molecular network medicine becomes a hot topic, and the molecular networks of some diseases have been built up with text mining and bioinformatic approaches [22]. These molecular networks could be useful for therapeutic mechanism elucidation or new drug discovery [23, 24]. However there was not enough information about the patients with TCM pattern expression for building up the molecular network of the TCM pattern in certain diseases; more data from clinical trials are urgently needed in future studies. 

The major limitation in this report is that only two patterns in RA (cold and hot) were discussed, due to their highly representative features of TCM patterns in RA. However, TCM patterns are diverse and complicated even in a well-established disease such as RA. For example, deficiency pattern is another common TCM pattern in RA. Deficiency pattern has a high incidence rate in elderly patients and among those with late-stage RA. Though the molecular network of the RA deficiency pattern is under construction, TCM pattern diagnosis is complicated allowing for the variety of patterns which exist and the fact that patients often present with a mixture of patterns. Designing an efficient way to study such complex TCM patterns remains an interesting and urgent issue. 

## 5. Conclusions

The biomedical combination therapy and TCM therapy were more effective in treating RA patients with TCM cold and hot patterns, respectively, and the mechanisms were partially in affecting the different pathways of the TCM pattern molecular networks in RA patients targeted by the pharmacological molecular networks of the interventions. All together, our results suggest that the mechanism in treating some diseases with corresponding TCM patterns could be explored by merging molecular pharmacological networks of the interventions and the networks of the disease with TCM corresponding patterns. 

## Figures and Tables

**Table 1:** Most related pathways affected by MTX + SZZ combination.

ID	Description	*P* value
6395	RNA splicing	2.15*E* − 196
6119	Oxidative phosphorylation	2.98*E* − 132
7165	Signal transduction	6.364*E* − 24
7264	Small GTPase mediated signal transduction	2.695*E* − 23
42773	ATP synthesis coupled electron transport	0.0005551
51436	Regulation of ubiquitin-protein ligase activity during mitotic cell cycle	3.178*E* − 19
9607	Response to biotic stimulus	2.927*E* − 05
7596	Blood coagulation	1.858*E* − 23
12501	Programmed cell death	4.023*E* − 11
7169	Transmembrane receptor protein tyrosine kinase signaling pathway	2.405*E* − 23
19	Regulation of mitotic recombination	3.914*E* − 11

**Table 2:** Most related pathways affected by TCM intervention.

ID	Description	*P* value
6260	DNA replication	8.87*E* − 33
8543	Fibroblast growth factor receptor signaling pathway	4.35*E* − 34
8633	Activation of proapoptotic gene products	4.39*E* − 15
12501	Programmed cell death	5.5*E* − 09
44249	Cellular biosynthetic process	6.15*E* − 11
7265	Ras protein signal transduction	1.27*E* − 08
7242	Intracellular signaling cascade	3.18*E* − 14
51242	Positive regulation of cellular process	3.42*E* − 13
51244	Regulation of cellular process	3.1*E* − 12
51252	Regulation of RNA metabolic process	7.83*E* − 09

**Table 3:** Two factors obtained in factor analysis after oblique PROMAX rotation*.

Symptoms	Factor 1	Factor 2
Cold feeling in whole body	0.710	
Cold feeling in joints	0.645	
Cold feeling in limbs	0.600	
Lassitude	0.580	0.280
Heavy feeling in limbs	0.514	0.445
Numbness in limbs	0.359	0.220
Vexation	0.334	0.544
Feverish joint		0.510
Thirst		0.457
Nocturia	0.260	0.358
Dizziness	0.240	0.347
Turbid yellow colored urine		0.331
Fever		0.297

*The data in the table are factor loadings obtained after oblique PROMAX rotation. The factor loading indicates the correlation power of the symptom with the factor. A loading value of more than 0.20 manifests a definite correlation of the symptom with the factor.

**Table 4:** TCM pattern changes after treatment for 24 week treatment.

	Pattern change	Case no.
TCM group	Cold dominant remains	49
	Cold dominant to noncold dominant	56
	Noncold dominant remains	75
	Noncold dominant to cold dominant	33

Biomedicine group	Cold dominant remains	57
	Cold dominant to noncold dominant	30
	Noncold dominant remains	84
	Noncold dominant to cold dominant	23

**Table 5:** Pathways involved in the intersection between TCM cold and hot patterns in RA.

ID	Description	*P* value
51436	Negative regulation of ubiquitin-protein ligase activity during mitotic cell cycle	3.14*E* − 78
31145	Anaphase-promoting complex-dependent proteasomal ubiquitin-dependent protein catabolic process	3.14*E* − 78
51352	Negative regulation of ligase activity	8.96*E* − 78
51444	Positive regulation of ubiquitin-protein ligase activity	8.96*E* − 78
51437	Positive regulation of ubiquitin-protein ligase activity during mitotic cell cycle	6.7*E* − 77
51443	Positive regulation of ubiquitin-protein ligase activity	1.76*E* − 76
51439	Regulation of ubiquitin-protein ligase activity during mitotic cell cycle	1.14*E* − 75
51351	Positive regulation of ligase activity	2.79*E* − 75
51438	Regulation of ubiquitin-prtein ligase activity	8.31*E* − 74
51340	Regulation of ligase activity	8.83*E* − 73

## Abbreviations

ACR: American College of Rheumatology
ATP: Adenosine triphosphate
BIND: Biomolecular Interaction Network Database
BiNGO: Biological Network Gene Ontology
BioGRID: The General Repository for Interaction Datasets
DIP: Database of Interacting Proteins
DMARDs: Disease modifying antirheumatic drugs
GMP: Good Manufacturing Practice
GO: Gene ontology
HPRD: Human Protein Reference Database
IntAct: Database system and analysis tools for protein interaction data
IPA: Ingenuity pathways analysis
LCA: Latent class analysis
MINT: Molecular Interactions Database
MTX: Methotrexate
NSAIDs: Nonsteroidal antiinflammatory drugs
PN: Panax notoginseng
PPI: Protein-protein interaction
RA: Rheumatoid arthritis
RNA: Ribonucleic acid
SB: Salvianolic acid B
SMPN: *Salvia miltiorrhiza* and *Panax notoginseng* in combination
SM: *Salvia miltiorrhiza*
SSZ: Sulfasalazine
TCM: Traditional Chinese medicine.
